# Enhancing the Adhesion of Polyaniline on Steel Substrates Without a Binding Agent: Evaluated by ASTM D 3359 Tape Test and Sodium Chloride (NaCl) Exposure

**DOI:** 10.3390/polym17081082

**Published:** 2025-04-17

**Authors:** Saleh Aldwais, Ali A. Al-Muntaser, Chen Chen, Jaqueline Robles, Anish Pal, Jeremiah T. Abiade

**Affiliations:** 1 Department of Mechanical and Industrial Engineering, University of Illinois, Chicago, IL 60607, USA; cchen215@uic.edu (C.C.); jrojas21@uic.edu (J.R.); apal8@uic.edu (A.P.); jta@uic.edu (J.T.A.); 2 Department of Physics, Faculty of Education and Applied Sciences at Arhab, Sana’a University, Sana’a P.O. Box 775, Yemen; almuntser2015@gmail.com

**Keywords:** adhesion enhancement, polyaniline, steel substrate, pre-surface treatment, characterization

## Abstract

This study presents a method for enhancing the adhesion of chemically synthesized polyaniline on steel substrates without the need for a binding agent. Hydrochloric acid (HCl) was used in the synthesis of polyaniline. The experiment details the in situ chemical synthesis of polyaniline and its application as a coating on steel surfaces using an air spray technique. Pre-surface treatment, including cleaning and sanding, was performed on the steel substrates prior to coating. Following the application of the polyaniline coating, heat treatment was applied, where the coating was heated to 350 °F for 3 h after it was fully dried. The adhesion properties of the coating were evaluated using the ASTM D 3359 adhesive tape test, along with short- and long-term exposure to 3.5% sodium chloride (NaCl) solution. Additional analysis, including SEM, XPS, XRD, and coating thickness measurements, demonstrates the effectiveness of polyaniline in enhancing adhesion on steel substrates.

## 1. Introduction

Polyaniline (PANI) is a conductive polymer with unique properties, making it a promising material for various technological and industrial applications. It was first discovered in the late 19th century, but its significance was not highlighted until 1980 when it was rediscovered by researchers. Polyaniline is known for its electrical conductivity, ease of synthesis, and the ability to modify its properties to suit different needs. For these reasons, chemists Hideki Shirakawa, Alan Heeger, and Alan MacDiarmid were awarded the Nobel Prize in Chemistry in 2000 for their development of conductive polymers, including polyaniline [1,2,3]. Polyaniline is a versatile material due to its ability to form protective coatings that shield metal surfaces from corrosion and environmental factors. It is used in many industrial applications, such as manufacturing, flexible electronics and supercapacitors, as it is a non-toxic, low-cost material, making it an ideal alternative to hazardous materials like lead. Economically, corrosion poses a significant threat, with an estimated annual cost of around USD 2.5 trillion, equivalent to 3.4% of the global GDP. More than 10% of the world’s total metal production is subjected to corrosion annually, making the adoption of preventive strategies essential [4,5,6,7,8].

One of the challenges in utilizing polyaniline as a protective coating is its poor adhesion to metal surfaces, particularly steel. Previous research has attempted to improve adhesion by incorporating various external bonding agents, including polymer binders, epoxy resins, thiokol rubber, silane coupling agents, and nanoparticles such as titanium dioxide [9,10,11,12,13]. While these agents can enhance adhesion, several studies have reported that their inclusion often reduces the active polyaniline content, which can negatively impact its intrinsic properties, such as electrical conductivity and corrosion resistance. For example, Pour-Ali et al. [10] and Radhakrishnan et al. [11] observed diminished conductivity and protective performance in coatings with high binder content. Therefore, we aimed to enhance adhesion without relying on such additives, preserving the functional benefits of pure polyaniline. Alternative surface preparation methods, such as plasma electrolytic polishing (PEP), have demonstrated promising improvements in adhesion and surface quality in previous studies [14,15]. However, mechanical polishing was selected for this study due to its simplicity, cost-effectiveness, and suitability for laboratory-scale experimentation.

To address this challenge, we developed a method that relies on precise surface preparation followed by thermal treatment to enhance adhesion. We conducted surface preparation processes that included cleaning the surface using an ultrasonic cleaner and sanding with 60, 80, and 180 grit sandpaper for gradual surface refinement. The subsequent cleaning steps involved using acetone, methanol, and IPA to ensure the removal of any residues or contaminants. We used the Branson 1510 ultrasonic cleaner with no heat, applying a 10 min sonication period after each step. Full details of this process will be discussed in the surface preparation section. After completing the surface pre-treatment, we applied polyaniline to the steel substrate via spraying. Once the coating dried at room temperature, it underwent thermal treatment at 350 °F (176.7 °C) for 3 h. The coating’s performance was evaluated using adhesion tests, including the ASTM D 3359 tape test, and exposure to 3.5% sodium chloride (NaCl). Characterization techniques such as SEM, XPS, XRD, and thickness measurements showed that optimal thermal conditions improved adhesion and coating quality. This research focused on improving polyaniline adhesion to steel through thermal treatment. By synthesizing polyaniline, preparing the surface, applying the coating, and then using thermal treatment, we demonstrated that the right temperature and duration are crucial to enhancing adhesion performance. Unlike previous studies that rely on external binding agents, this work demonstrates that proper surface preparation and controlled heat treatment alone can yield strong adhesion of pure polyaniline to steel substrates. This highlights the potential of a binder-free polyaniline coating approach using thermal treatment and surface preparation alone, offering a simpler and cost-effective alternative.

## 2. Materials and Methods

### 2.1. Materials

**Figure 1:**
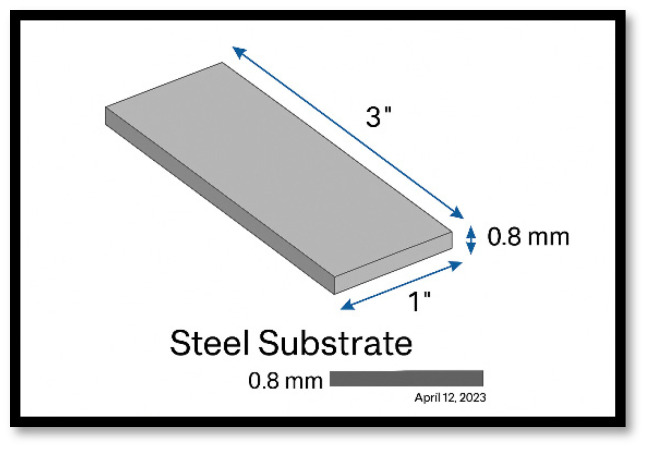
Steel substrate (low-carbon steel, AISI 1010/1008, 3” × 1” × 0.8 mm).

All materials used in this study were ordered from Thermo Fisher Scientific (Waltham, MA, USA), including Aniline (A14443 Aniline, 99+%), 1 N HCl (Hydrochloric acid, 36%), DI water, and ammonium persulfate (A10533-0B Ammonium peroxydisulfate, 98%), as well as an ice bath. The other tools utilized in the preparation process included beakers, a centrifuge set at 5000 rpm, a temperature sensor, and an air gun (Master Airbrush from Home Depot, Chicago, IL, USA). The steel substrate used was uncoated low-carbon steel (Grade: AISI 1010/1008), commercially available as ‘PLAIN STEEL’, part number 325027, manufactured by EVERBILT and purchased from Home Depot (Chicago, IL, USA). The steel sheet was cut in the laboratory into samples measuring precisely 3 inches by 1 inch, with a thickness of 0.0315 inches (0.800 mm), and an average weight of 11.585 g, as depicted in Figure 1. All reagents were used as received.

### 2.2. Polyaniline Synthesis

**Figure 2:**
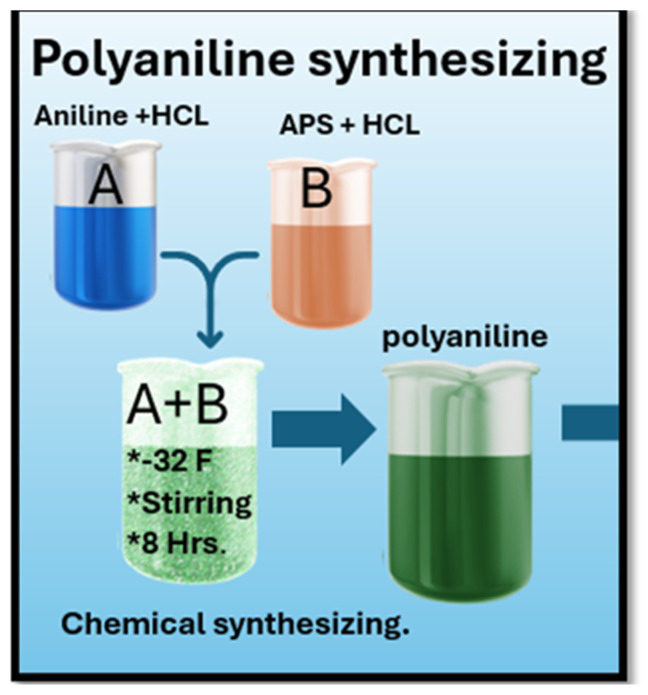
Experimental setup for polyaniline synthesis. Chemical method.

The process for the synthesis of polyaniline is shown in Figure 2, which is based onhe same process as reference [16]. The conducting polymer polyaniline (PANI) was synthesized via chemical oxidation of aniline in an acidic medium, employing ammonium persulphate as the oxidizing agent. In the synthesis process, a solution was prepared by mixing 20 mL of 1 N HCl (prepared by dilution of concentrated hydrochloric acid (36%) with deionized water) with 180 mL of deionized (DI) water, and 2.7 mL (0.03 mol) of aniline was dissolved in the acidic solution. The temperature of the resulting solution was maintained at 0 to 5 °C, which is 32 F. The temperature was reduced using an ice bath. Subsequently, a pre-cooled ammonium persulphate aqueous solution was slowly added (over 90–120 min) to the acidic aniline solution. After allowing the reaction mixture to stand at a low temperature (approximately 0 to 5 °C) for 12 h, a greenish-black precipitate formed.

### 2.3. Pretreatment Surface on Substrate Approach

**Figure 3:**
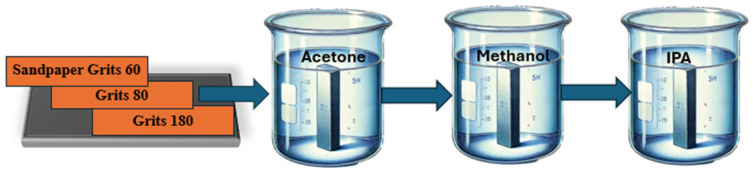
Substrate surface preparation approach.

The substrate chosen is steel due to its industrial applications and susceptibility to corrosion. As in Figure 3, we started with a polishing procedure using sandpaper with grits of 60, 80, and 180 for gradual surface refinement. The subsequent cleaning steps involved Acetone, Methanol, and IPA, ensuring the removal of any residues and contaminants. A crucial aspect of our methodology was the use of ultra-sonication with a Branson 1510 instrument, specifically set with sonics and no heat. Each step in the process was followed by a 10 min ultra-sonication period, contributing to the efficacy of the cleaning and preparation steps.

### 2.4. Coating Process (Air Spray)

**Figure 4:**
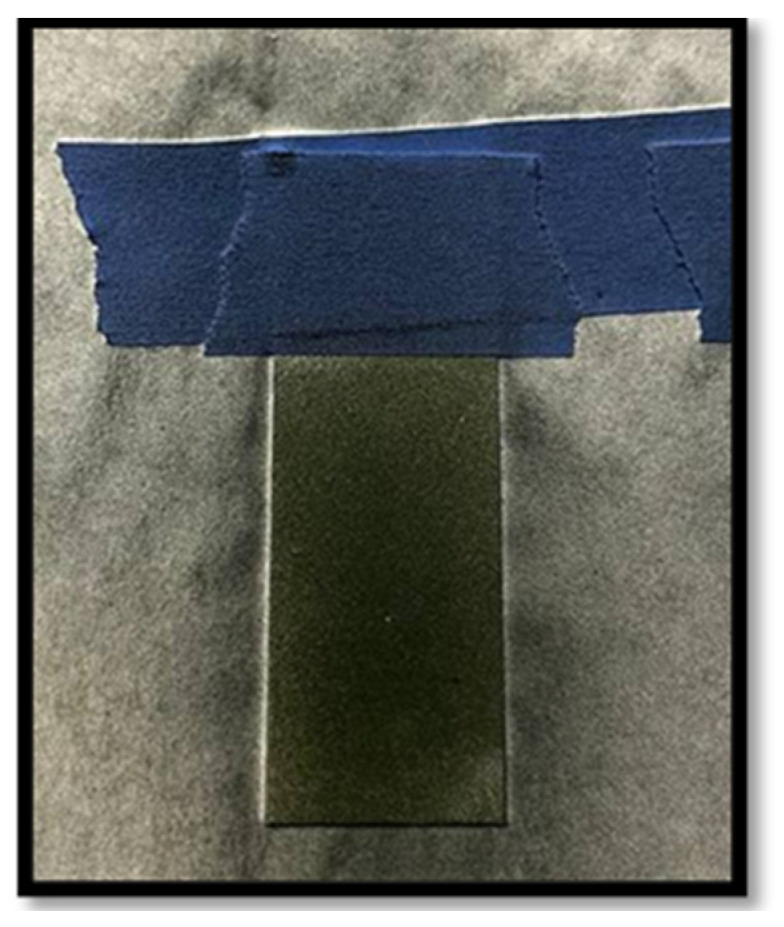
Polyaniline coated on steel.

**Figure 5:**
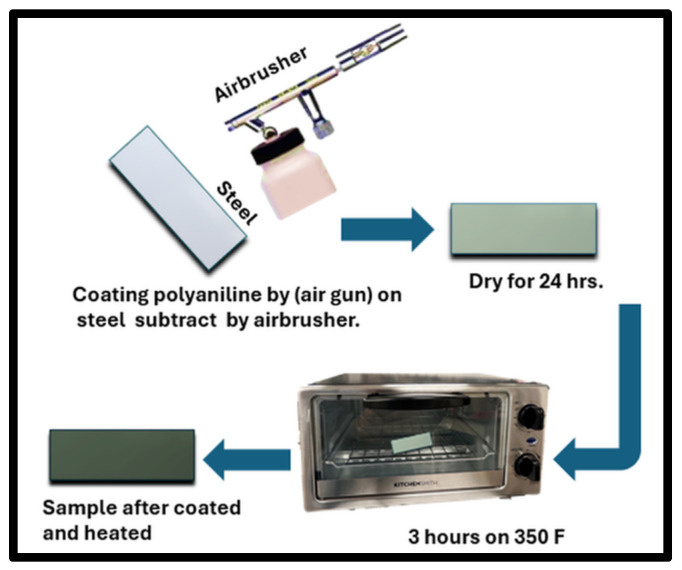
Coating and heating process of the prepared samples.

The coating here was applied by an airbrush gun [12,17,18,19]. The polyaniline solution was loaded into the airbrush gun, and we coated the steel face shown in Figure 4; the top part was taped as shown in Figure 5 and remained clean after coating, Weconducted 4 to 5 coating rounds to achieve the desired coverage. Following the application, the coated samples were left to cure for 12–24 h in a room temperature with clean air. The resulting coating exhibited a distinctive dark green color, and the average thickness ranged between 22 and 38 μm.

### 2.5. Fabrication (Heat Treatment)

Instead of traditional adhesion agents, heat treatment was explored as an alternative approach in the fabrication process. As mentioned in the introduction, binding agents are commonly added to improve adhesion, as evidenced by H. Wang et al. [13], A. Arshak et al. [9], Pour-Ali et al. [10], and S. Radhakrishnan et al. [11]. These studies explore various binding agents, such as polymer binders, epoxy resins, and Thiokol rubber, which can improve adhesion but often result in increased costs and processing complexity. Additionally, in some cases, the materials added to polyaniline made up more than 50% of the compound, thereby reducing the effectiveness of polyaniline itself.

We tested multiple samples at different heat treatment temperatures to evaluate the efficacy of this method. After numerous attempts, we determined that 350 °F for 3 h in an open oven was the optimal heat treatment for our experiment. Adhesion testing was conducted on all samples to verify the effectiveness of this method. The results of these tests will be thoroughly discussed in the heat treatment analysis results.

## 3. Testing

### 3.1. NaCl Adhesion Test of Polyaniline-Coated Steel Samples

**Figure 6:**
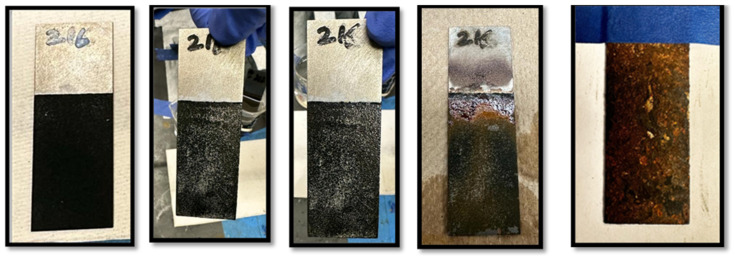
Sample 1: No post-coating heat treatment. Note: All sub-figures in this panel represent the same sample at different stages during the NaCl exposure test. The sample was not changed throughout the process; variations in appearance are due to corrosion progression. The final sub-figure on the right shows the condition of the sample after 90 days of NaCl exposure, with corrosion progression clearly visible.

**Figure 7:**
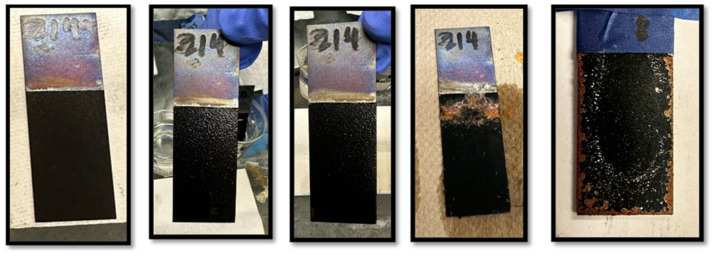
Sample 2: Post-coating heat treated for 3 h at 350 °F. Note: All sub-figures in this panel represent the same sample labeled “214” during the NaCl exposure timeline. The sample remained consistent; the visual changes are due to localized corrosion effects over time. The final sub-figure on the right reflects the sample condition after 90 days, showing the protective effect of heat treatment against corrosion.

Two polyaniline-coated steel samples, Sample A and Sample B, were tested for adhesion in a 3.5% NaCl solution (50 mL deionized water) to assess corrosion resistance over time, with weekly replenishment of the solution to counter evaporation. The samples remained at room temperature throughout, with adhesion and surface condition checks on days 5, 15, 30, 90, and finally day 100, when they were sanded with grade 60 sandpaper to evaluate coating integrity. Sample A, depicted in Figure 6, received no post-coating heat treatment, while Sample B (Figure 7) was heat-treated for 3 h at 350 °F. Observations over these intervals provided insight into the effectiveness of heat treatment on coating durability in saline conditions. Samples were weighed only before initiating the NaCl test. Photographic documentation was performed on days 5, 15, 30, and 90. The test, initially planned for 90 days, was unintentionally extended to 100 days due to scheduling constraints. On day 100, coatings were intentionally removed to examine the substrate surfaces. Frequent replenishment of evaporated NaCl solution every 5–7 days may have altered surface conditions, rendering post-test weight measurements unreliable for accurately quantifying corrosion.

### 3.2. Post-Cleaning Surface Comparison (Day 100)

**Figure 8:**
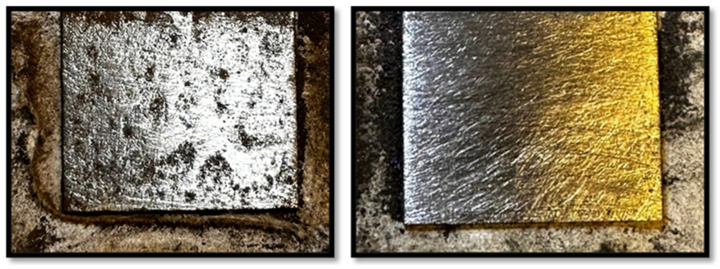
Sample 1 (**left**) and Sample 2 (**right**) after cleaning on day 100.

Figure 8 shows the surface conditions of Samples 1 and 2 after cleaning with grade 60 sandpaper on day 100. These images visually compare the effectiveness of the polyaniline coatings after prolonged exposure to NaCl.

### 3.3. Adhesion Test by ASTM D 3359 Adhesive Tape

**Figure 9:**
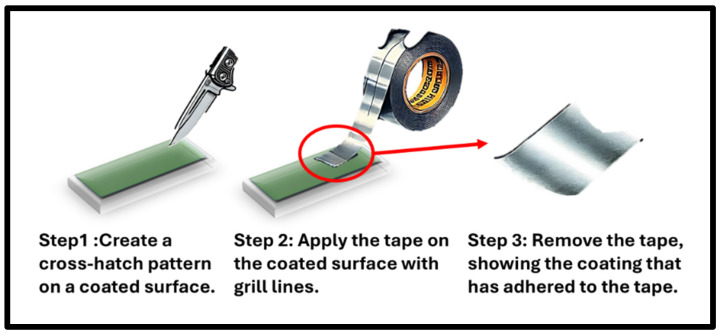
Adhesion test process ASTM D 3359 adhesive tape.

ASTM D 3359 Tape is a standard adhesive test method [20,21,22] that involves the use of adhesive tape to assess the adhesion strength of coatings on substrates. In this method, a specified pressure-sensitive tape is applied to the coated surface and then swiftly removed, evaluating the degree of coating adhesion. The tape’s adhesive properties provide a standardized means to gauge the coating’s resistance to detachment or peeling, offering valuable insights into the quality and durability of the applied coatings on various materials. In Figure 9, provides a simpler way to understand the test process. This method plays a crucial role in quality control processes across industries where coating adhesion is a critical factor. In this test, two new samples, Samples 1 and 2, have been introduced. Sample 1 features a coating thickness of 20 μm to 38 μm, coated with polyaniline and then heated for 3 h at 350 °F. Similarly, Sample 2 underwent the same coating process as Sample 1 but was not heated after coating. These variations in heat treatment provide an opportunity to explore the influence of this parameter on the adhesion properties of the coatings in subsequent testing.

## 4. Results and Discussion

### 4.1. Post-Cleaning Surface Comparison (Day 100)

Figure 8 illustrates the surface conditions of Sample 1 and Sample 2 after cleaning with grade 60 sandpaper on day 100, providing a visual comparison of the effectiveness of polyaniline coatings following prolonged NaCl exposure. Sample 1, after cleaning, displayed extensive corrosion and significant coating loss, revealing large corroded areas with some metal loss due to severe degradation from the extended NaCl exposure. The steel substrate appeared rough and uneven, suggesting weak adhesion of the coating that allowed corrosion and metal erosion to progress. Further SEM analysis will be conducted to quantify the degree of corrosion and examine the surface morphology in greater detail. In contrast, Sample 2 maintained most of its original smooth steel surface, closely resembling its condition before NaCl exposure, with the coating remaining well adhered and only minor areas of loss observed. The steel surface remained largely intact, with no significant corrosion or metal loss detected. SEM analysis will verify the preservation of Sample 2’s steel substrate and will identify any microscopic defects not visible in the current images.

### 4.2. Adhesion Test Analysis Using ASTM D 3359 Tape

The results obtained for the prepared samples using ASTM D 3359 adhesive tape were summarized in Table 1. Sample 1 was subjected to heat treatment of 3 h at 350 °F and exhibited notable improvements in adhesion based on the ASTM D 3359 adhesive tape test. The results indicated enhanced adhesion, with an approximate 5% loss from the coating, reflecting a positive outcome. Conversely, Sample 2 was devoid of any heat treatment. The absence of heat treatment resulted in significant adhesion deterioration, evidenced by a substantial loss of around 50% of the coating, as clearly depicted in the test pictures. Collectively, the outcomes of Sample 1 underscore the pivotal role of heat treatment in improving adhesion, highlighting the significant impact of this parameter on the integrity of the coatings. The measurement is estimated to classification of adhesion from Table 2, and the same measurement classification used in [22,23,24].

### 4.3. The Heat Treatment Temperature Analysis

**Figure 10:**
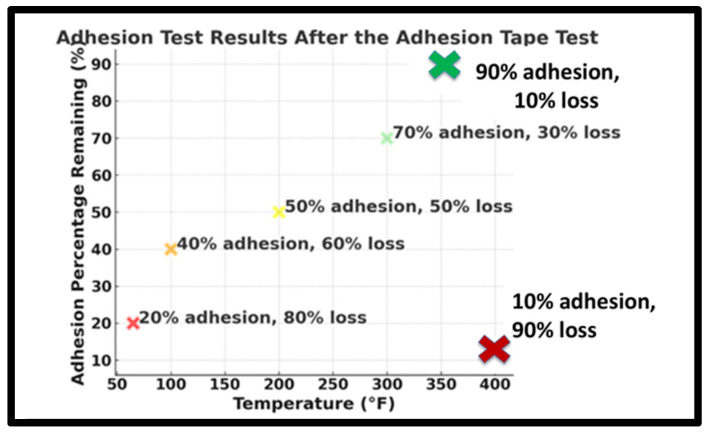
Heat Treatment Temperature Analysis. Note: “Adhesion performance (%)” indicates the Sestimated percentage of coating remaining or lost after the ASTM D 3359 tape test, based on visual evaluation.

As mentioned earlier, 350 °F was determined to be the optimal temperature for our experiment, as illustrated in Figure 10. This conclusion was reached after testing samples at various temperatures, starting from room temperature and increasing up to 420 °F. During this process, we carefully considered the thermal degradation temperatures of both steel and polyaniline to ensure material integrity. Steel typically melts at temperatures between 2500 °F and 2800 °F depending on the alloy composition [25]. Polyaniline, however, begins to degrade thermally at approximately 400 °F. According to Lee and Char [26], the decomposition of PANI’s backbone chains begins at this temperature, leading to significant weight loss in thermogravimetric analysis (TGA) and differential thermogravimetric analysis (DTG) curves. In our study, we observed a marked decline in adhesion performance as the temperature approached 400 °F, confirming that exceeding this threshold compromises PANI’s structural integrity. To maintain optimal adhesion while preventing thermal degradation, we found that 350 °F provides the best balance between performance and thermal stability. Adhesion tests at this temperature consistently outperformed those conducted at higher temperatures. The graph in Figure 10 shows the results where green areas indicate higher adhesion and red areas indicate lower adhesion. As noted, adhesion performance dropped significantly when the temperature exceeded 400 °F, as the coating began to degrade and lose its adhesive properties.

Note: In this study, all heat-treated samples were exposed to 350 °F for a consistent duration of 3 h. This duration was selected to allow sufficient molecular rearrangement within the polyaniline coating while avoiding thermal degradation. Based on our experimental results, this fixed duration proved effective in improving adhesion performance. However, future research could explore the effect of varying heat treatment durations (e.g., 1 h or 5 h) to further optimize adhesion without compromising the structural integrity of polyaniline.

### 4.4. Scanning Electron Microscope (SEM)

**Figure 11:**
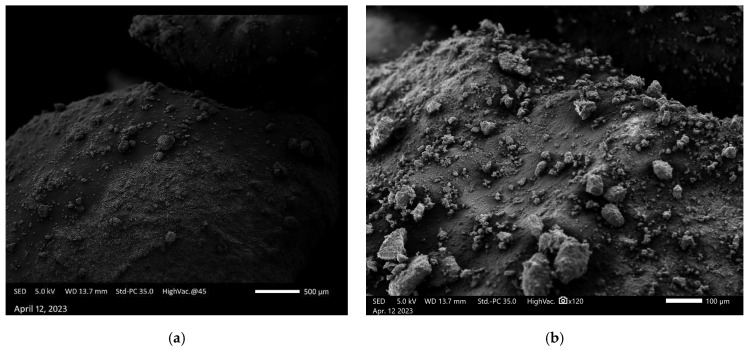
SEM of standard polyaniline at (**a**) 500 μm and (**b**) 100 μm.

**Figure 12:**
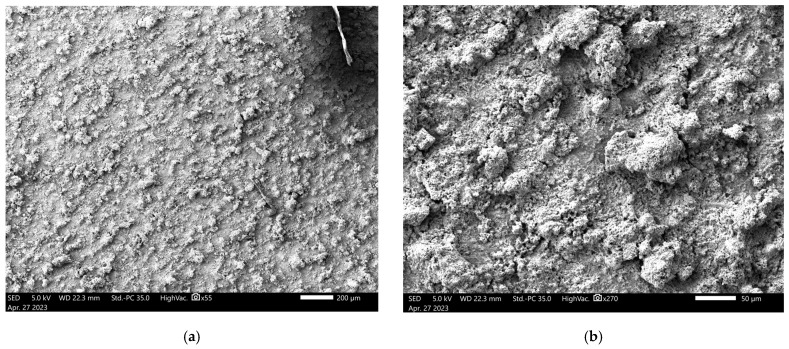
SEM of heated sample at (**a**) 200 μm and (**b**) 50 μm.

**Figure 13:**
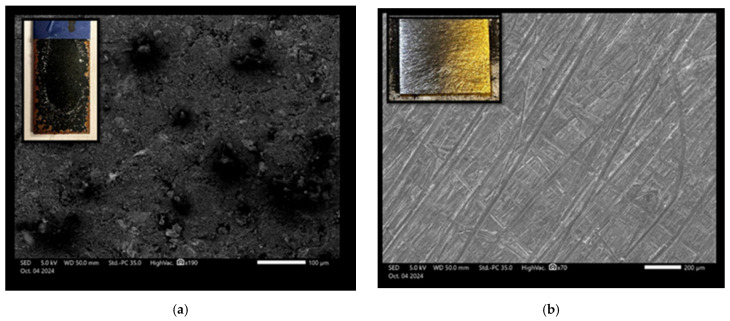
SEM images of the heated sample of PANI coating steel surface (**a**) after 100 days in NaCl exposure. (**b**) The same sample after 100 days of NaCl exposure, post-cleaning, and sandpaper removal.

**Figure 14:**
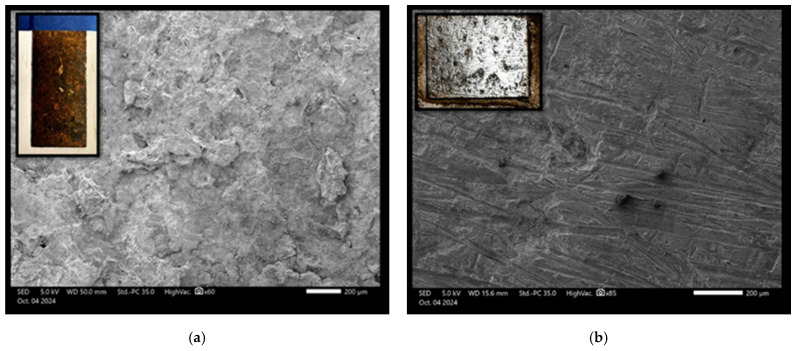
SEM of non-heated sample of PANI coating steel surface at 200 mm (**a**) after 100 days in NaCl exposure and (**b**) the sample after 100 days in NaCl exposure, post-cleaning, and sandpaper removal.

The scanning electron microscope (SEM) images were captured using the JEOL JSM IT500 HR (JEOL Ltd., Tokyo, Japan) at the Research Resources Center lab at the University of Illinois at Chicago, to observe the surface morphology, adhesion, and interaction between polyaniline (PANI) and a steel substrate under various experimental conditions. Standard polyaniline powder, acquired from Thermo Fisher Scientific (Product ID: 042581.06), was imaged at magnifications of 500 µm and 100 µm, as shown in Figure 11a,b, respectively, revealing its granular morphology with irregularly shaped particles with varying sizes and textures, which served as a baseline for comparison with coated samples. When a PANI-coated steel substrate was heated at 350 °F for 3 h, as shown in Figure 12a,b at 200 µm and 50 µm magnifications, the SEM images displayed a smooth and continuous PANI layer that covered the steel surface uniformly, free from visible interruptions. After this heated sample was exposed to NaCl for 100 days, SEM images (Figure 13a) at 100 µm magnification revealed that the PANI coating remained largely intact, with minor surface changes but overall good coverage. Following this NaCl exposure, the surface was cleaned, and the PANI coating was completely removed using grade 60 sandpaper, as seen in Figure 13b at 200 µm magnification, revealing a smooth, clean steel surface. In contrast, a non-heated PANI-coated steel sample also exposed to NaCl for 100 days exhibited significant surface corrosion and degradation, with the PANI layer partially degraded, exposing the steel to further corrosion, as shown in Figure 14a at 200 µm magnification. After cleaning, the PANI was fully removed from the non-heated sample using sandpaper, as shown in Figure 14b at 200 µm magnification, revealing a corroded and rough steel surface, which demonstrated the extent of NaCl-induced deterioration in the absence of heat treatment.

### 4.5. X-Ray Photoelectron Spectroscopy (XPS)

**Figure 15:**
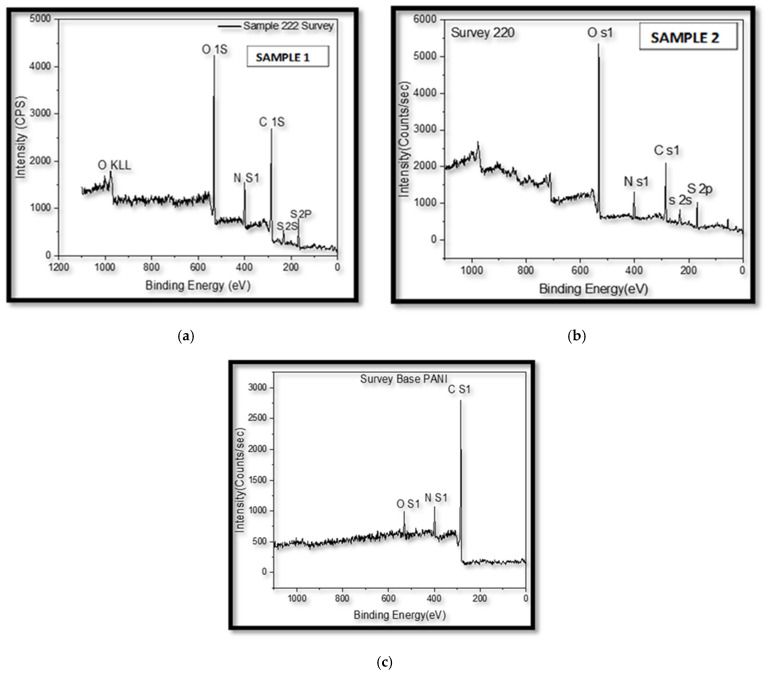
XPS analysis results for (**a**) Sample 1: coated with polyaniline (PAIN) and heat-treated, (**b**) Sample 2: coated with polyaniline (PAIN) without heat treatment, and (**c**) Standard base polyaniline obtained electrochemically.

X-rayhotoelectron spectroscopy (XPS) is an analytical technique that provides information about the elemental composition, chemical state, and electronic structure of materials by analyzing the energy of electrons emitted when the sample surface is irradiated with X-rays. In this study, XPS analysis was conducted 30 days after the samples were coated to determine their surface composition, with testing carried out at the Research Resources Center laboratory at the University of Illinois Chicago. The analysis identified key elements, including O1s, N1s, C1s, S2s, and S2p states that indicate interactions between polyaniline and the steel substrate. In Figure 15a, Sample 1, a steel substrate coated with polyaniline with a thickness ranging from 20 to 38 µm, was subjected to a three-hour heat treatment at 350 °F. XPS analysis reveals the presence of O1s, N1s, C1s, S2s, and S2p. It is noteworthy that the O KLL in XPS is regarded as an error in both samples. Sample 1 exhibits a lower O1s and higher C1s content, attributed to the implemented heat treatment. Note that I performed the XPS 30 days after the coating was applied. In XPS, the binding energy peaks are used to identify the elements present in a sample. The “O KLL” refers to oxygen, and KLL indicates the specific energy level (shell) associated with the electron transitions in the XPS spectrum.

In contrast, Sample 2, shown in Figure 15b, is XPS for the same sample in the previous adhesion test (part of the sample was taken to fit XPS) a steel substrate coated with polyaniline, shares a similar coating thickness of 20 to 38 µm. However, no heat treatment is applied, and polyaniline synthesis is achieved using a chemical method on site. XPS analysis confirms the presence of O1s, N1s, C1s, S2s, and S2p. Sample 2 shows higher O1s and lower C1s content, indicating the influence of the absence of heat treatment. Figure 15c displays a standard base polyaniline sample obtained as a powder through an electrochemical synthesis ordered from a commercial source, which reveals the presence of O1s, N1s, and C1s. In comparison with Sample 1, the standard base polyaniline exhibits lower O1s and higher C1s content, a difference attributed to the heat treatment applied in Sample 1. Note: This standard polyaniline is created by electrochemical synthesis which is different from the chemical synthesis that we used in the lab, and this may affect the difference between Samples 1 and 2 compared to the standard in XPS.

**Figure 16:**
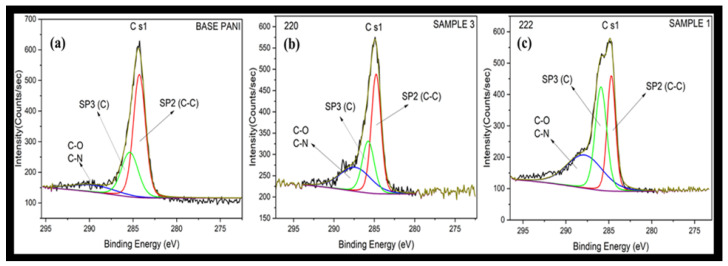
Fitting analysis of C s1 XPS for (**a**) Base, (**b**) Sample 2, no heat, and (**c**) Sample 1, heated.

Analysis of the carbon electron energy levels in each sample’s X-ray Photoelectron Spectroscopy spectra, shown in Figure 16a for the standard base, Figure 16b for Sample 2, and Figure 16c for Sample 1, revealed three distinct peaks associated with various types of carbon bonding [27]. Sample 1 (Figure 16c), consisting of a steel substrate coated with polyaniline synthesized through a chemical method with a subsequent 3 h heat treatment at 350 °F, exhibited the first peak with a binding energy of 284.9 eV, corresponding to (sp2) hybridization of C-C bonds, a second peak at 285.9 eV, representing C (sp3) hybridization, and the third peak with a binding energy of 287.9 eV, representing both C-O and C-N component. Also, in Sample 1 (Figure 16c), coated with polyaniline but lacking heat treatment, the first peak appeared at 284.5 eV representing (sp2) hybridization of C-C bonds, the second peak at 286 eV is related to C (sp3) hybridization, and the third peak at 287.5 represents both C-O and C-N component. In the third sample (Figure 16b) synthesized using an electrochemical method and serving as a standard reference (Figure 16a), the first peak at 283.4 eV represents (sp2) hybridization of C-C bond, the second peak at 285.3 eV is related to C (sp3) hybridization, and the third peak at 288.25 eV represents both C-O and C-N component.

### 4.6. X-Ray Diffraction (XRD) Analysis

**Figure 17:**
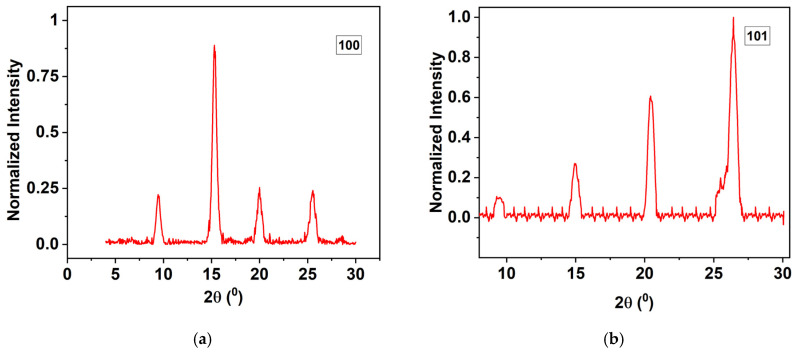
XRD polyaniline for sample with numbering of (**a**) 100 and (**b**) 101.

XRD analysis was conducted using the Bruker D8 Discover X-ray diffraction tool at the University of Illinois at Chicago’s Nanotechnology Core Facility, under the Research Resources Center, to investigate the crystalline structure of polyaniline before and after thermal treatment. Two samples were analyzed, with the XRD pattern labeled “100” representing unheated polyaniline and “101” representing polyaniline after thermal treatment as shown in Figure 17a,b, respectively. The “101” sample (Figure 17a) was taken from a steel substrate coated with polyaniline and analyzed 48 h after coating; minor contamination from the steel surface or airborne particles might have been present, but it was not expected to impact the results significantly. The XRD patterns exhibit distinct peaks associated with specific crystallographic planes, reflecting the structural organization of the polyaniline chains. Post-heating, changes in peak positions and intensity were observed as observed in Figure 17b, indicating an enhancement in crystallinity due to thermal effects. Comparing these results with the XRD pattern of polyaniline described in Article [28] confirms consistency in the crystalline structure before and after heating, with significant changes post-treatment. A peak around 2θ = 15°, attributed to periodic spacing within the polyaniline chains, remained after heating, though slight shifts in position and intensity suggested altered molecular ordering and increased crystallinity. A peak at approximately 2θ = 20°, indicating interchain spacing, was present in the untreated sample and showed changes post-heating, reflecting a potential phase transition. The peak near 2θ = 25°, associated with π–π stacking interactions between aromatic rings, was affected by heating, likely due to reorganization of these interactions, enhancing crystalline order. Additionally, a peak around 2θ = 26°, correlated with highly ordered crystalline domains, intensified after thermal treatment, suggesting enhanced crystallinity or a phase change. Peaks at lower 2θ values, typically indicating larger d-spacings and the long-range structural order, also exhibited shifts after heating, implying alterations in the structural arrangement and an increase in crystallinity or new phase formation in the polyaniline structure.

## 5. Correlation of Crystallinity and Surface Chemistry with Adhesion Performance

To better understand the enhanced adhesion observed in heat-treated samples, a correlation between crystallinity (from XRD) and surface chemistry (from XPS) is essential. The XRD analysis revealed that thermal treatment at 350 °F increased the crystallinity of the polyaniline coating, as shown by sharper and more intense diffraction peaks—particularly around 2θ = 25° and 26°, indicating improved π–π stacking and ordered domains. This enhancement in crystalline structure suggests a denser, more uniform film with fewer defects and better interfacial contact with the steel substrate.

Complementing this, the XPS results demonstrated a relative increase in carbon species (C1s) and a reduction in oxygen species (O1s) in heat-treated samples. This indicates a lower level of surface oxidation and a more stable chemical state, which likely improves cohesion within the polymer matrix and adhesion to the metal substrate. The higher presence of C–C (sp^2^) bonding in the heated sample, along with C–N interactions, reflects stronger and more interconnected molecular structures.

Together, these results support the hypothesis that thermal treatment improves both the structural and chemical characteristics of polyaniline coatings, contributing significantly to the observed enhancement in adhesion, as verified by both NaCl and ASTM D 3359 tape tests.

## 6. Conclusions

This study demonstrated that post-coating heat treatment significantly improves the adhesion of polyaniline on steel substrates. Two tests were conducted: a 100-day NaCl exposure followed by surface cleaning and the ASTM D 3359 adhesive tape test. In both cases, heat-treated samples showed superior adhesion compared to non-treated samples. The NaCl test revealed better surface quality in heat-treated samples after cleaning, while the ASTM D 3359 test showed only 5% coating loss for the heat-treated samples versus 50% loss for non-treated samples. Supporting data from SEM, XRD, and XPS further confirmed that heat treatment, along with the correct coating thickness, temperature, and duration of heating, plays a crucial role in enhancing adhesion without the need for adhesion agents. These findings offer a practical approach to improving the performance of polyaniline coatings on steel substrates.

## Figures and Tables

**Table 1:** The obtained results of ASTM D 3359 adhesive tape.

Sample Number	Before ASTM Test	After ASTM Test	Tape Result
Sample 1: Heated: 3 h at 350 °F. Tape Test Results: ➢ *Approximately 5% loss from the coating*	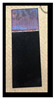	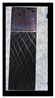	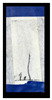
Sample 2: Heated: No heat treatment applied. Tape Test Results: ➢ *Approximately 50% to 60% loss from the coating*	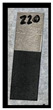	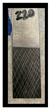	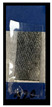

**Table 2:** The classification of adhesion test results.

Classification of Adhesion Test Results		
Classification	Percent Area Removed	Surface of Cross-Cut Area from Which Flaking Has Occurred for Six Parallel Cuts and Adhesion Range by Percent
5B	None (0%)	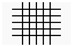
4B	Less than 5%	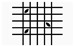
3B	5–15%	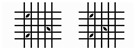
2B	15–35%	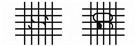
1B	35–65%	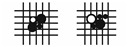
0B	Greater than 65%	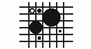

## Data Availability

The original contributions presented in this study are included in the article. Further inquiries can be directed to the corresponding author.
